# Picosecond pulsed laser illumination: an ultimate solution for photonic versus thermal processes’ contest in SOI photo-activated modulator

**DOI:** 10.1038/s41598-021-04710-w

**Published:** 2022-01-28

**Authors:** David Glukhov, Zeev Zalevsky, Avi Karsenty

**Affiliations:** 1 Advanced Laboratory of Electro-Optics (ALEO), Department of Applied Physics/Electro-Optics Engineering, Lev Academic Center, 21 Havaad Haleumi St., POB 16031, 9116001 Jerusalem, Israel; 2 Nanotechnology Educational and Research Center, Lev Academic Center, 9116001 Jerusalem, Israel; 3 Faculty of Engineering, Bar-Ilan University, 5290002 Ramat Gan, Israel; 4 Nanotechnology Center, Bar-Ilan University, 5290002 Ramat Gan, Israel

**Keywords:** Optics and photonics, Nanoscience and technology, Nanoscale devices

## Abstract

The functionality of a nanoscale silicon-based optoelectronic modulator is deeply analyzed while it appears that two competing processes, thermal and photonic, are occurring at the same time, and are preventing the optimization of the electro-optics coupling. While an incident illumination-beam first process is translated into photons, generating pairs of electrons–holes, a second process of thermal generation, creating phonons enables a loss of energy. Complementary studies, combining strong analytical models and numerical simulations, enabled to better understand this competition between photonic and thermal activities, in order to optimize the modulator. Moreover, in order to prevent unnecessary heating effects and to present a proposed solution, a picosecond pulsed laser is suggested and demonstrated as the ultimate solution so no energy will be wasted in heat, and still the photonic energy will be fully used. First ever-analytical solution to the heating produced due to the laser illumination applied on a nano-photonic device, while the illumination is produced in a periodic time changing function, e.g. a pulsed illumination, is presented. The present case study and proposed adapted solution can serve as a basis of generic approach in sensors’ activation towards optimized photonics absorption.

## Introduction

Coupling of physical properties has always been a preferred domain of interest for researchers and engineers, not only because of the multi-disciplines challenge, but also because it enables the study and application of combined properties in materials and devices, and of input–output conversion of signals. One well-known example is the electro-optic (EO) domain, in which EO devices are desirable for their ability to convert electrical input signals into light emission output, i.e. electroluminescence (EL) effect, or to transform incident illumination beam of absorbed photons into generated holes–electrons pairs, i.e. photoluminescence (PL) effect. Smart devices coupling combined properties, like optical and electrical (opto-electronic) or optical and thermal (opto-thermic), are well desirable in order to develop new advanced technologies. However, some strong competition may appear in the same device between several mechanisms, existing at the same time. This kind of contest between activation mechanisms becomes crucial when the competition remains between thermal and photonic generations. Unnecessary heat can cause malfunctioning or can prevent optimization. This is why thermal generation mainly interested researchers and engineers. General models for optical heating and thermal management in semiconductors were already presented four decades ago^1^. With time, textbooks addressed and treated generation and recombination processes of carriers due to thermal and photonic activation^2^. In addition, more specific models for heat generation and transport as well as for coupling of optical, electrical and thermal models were conducted for several types of devices such as transistors^3,4^, resonators^5^, solar cells^6^, light emitting devices (LED)^7^, organic light emitting devices (OLED)^8^, Vertical Cavity Surface-Emitting Lasers (VCSEL)^9^, lasers^10^, and fiber optics^11^. When compared to above specific devices, for which relevant models were developed accordingly, a new analysis was now required for the Silicon-On-Insulator Photo-Activated-Modulator (SOIPAM) device, sharing a V-groove aperture for illumination trigger, and enabling several activation mechanisms, as described below.

## Device special structure and mechanisms

### Photo-activated modulator (PAM)

**Figure 1:**
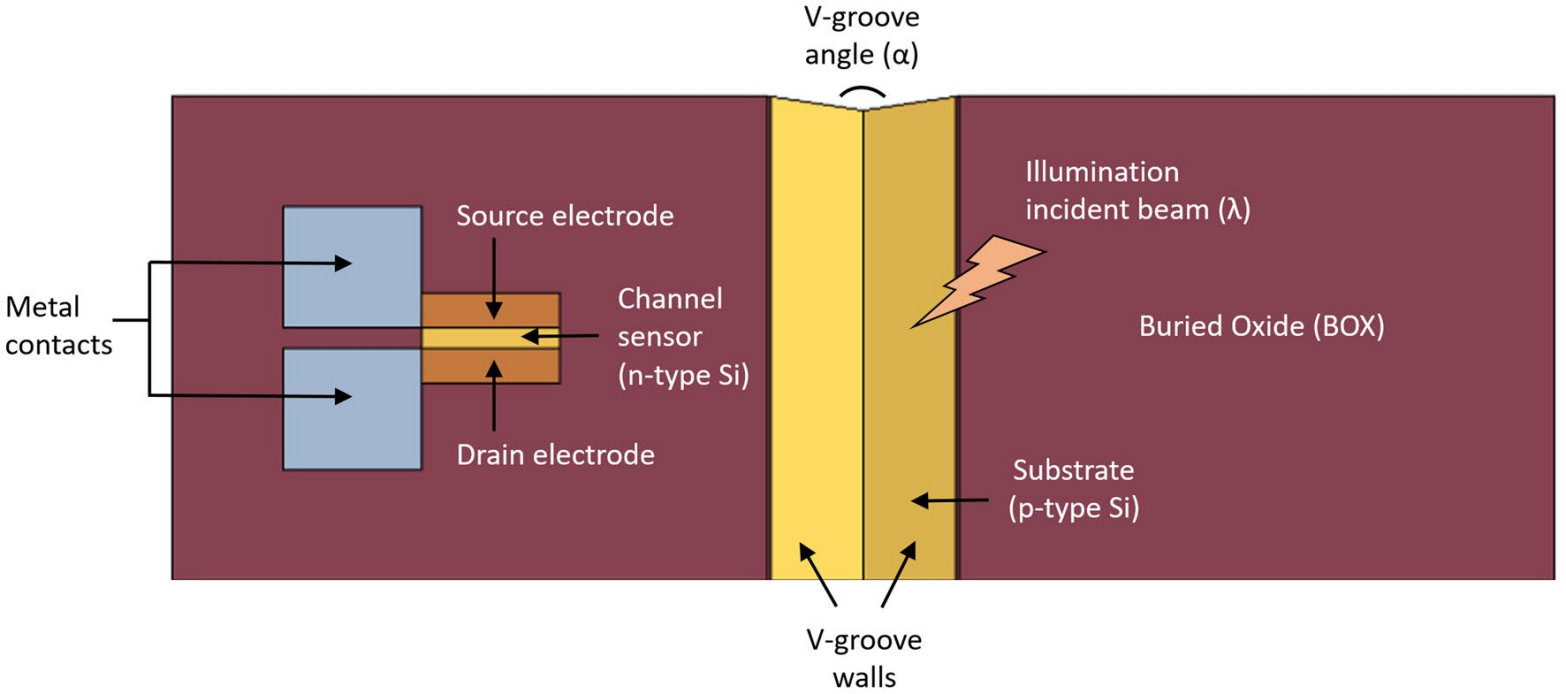
SOIPAM device 2D structure top view.

The Silicon-On-Insulator Photo-Activated-Modulator (SOIPAM) device is an electro-optical modulator so that the signal passing through it is electric (current) but is controlled by illumination (Fig. 1). When the device is not illuminated—the current is transmitted (i.e. normally ON), and when there is some incident illumination—it is stopped (OFF). SOIPAM structure’s top views, cross-section views, and mesh views were obtained using Comsol Multi-Physics software^12^.

**Figure 2:**
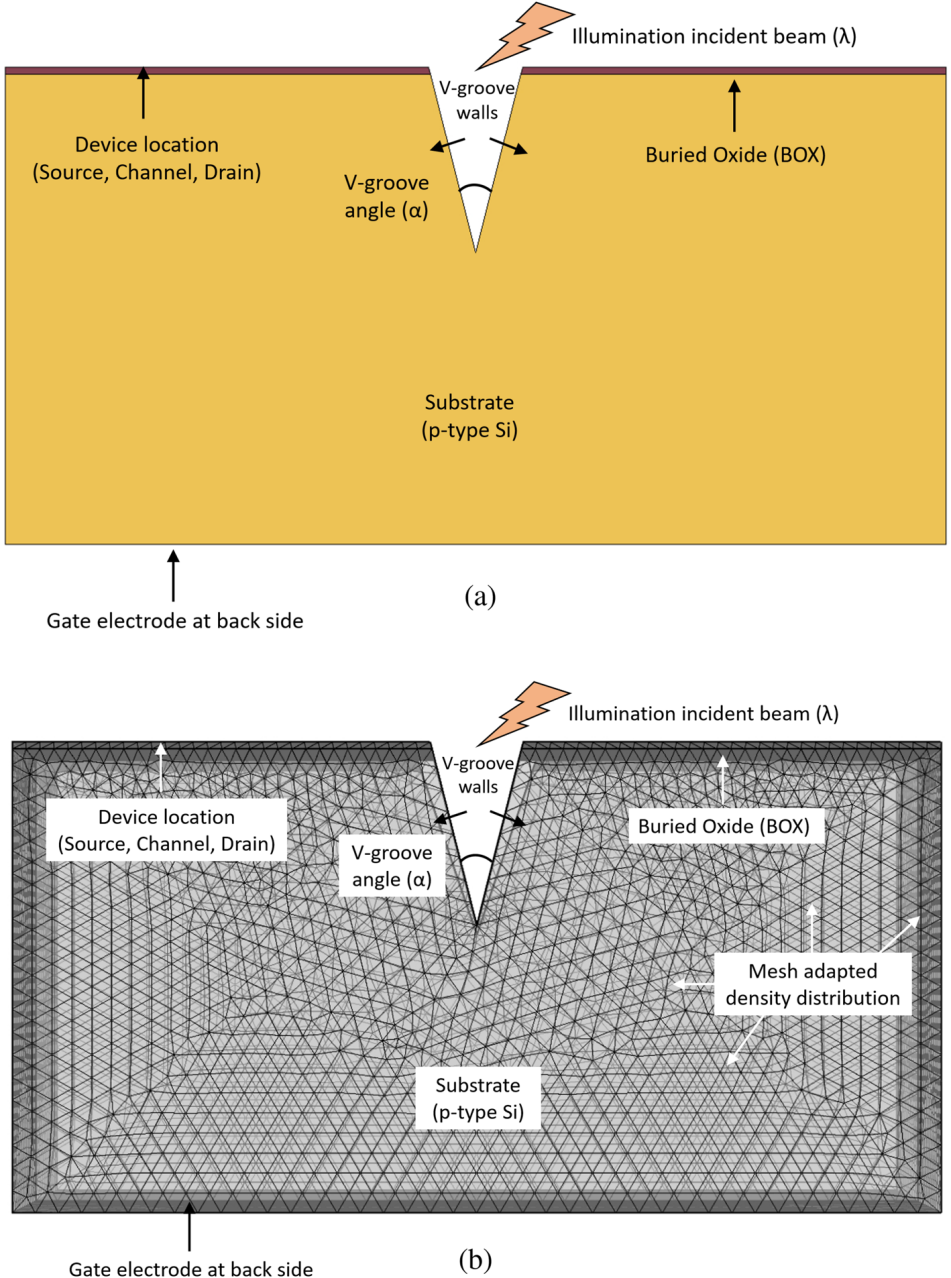
SOIPAM device 2D structure cross views. (**a**) 2D structure cross-section; (**b**) 2D mesh distribution showing finite elements.

**Figure 3:**
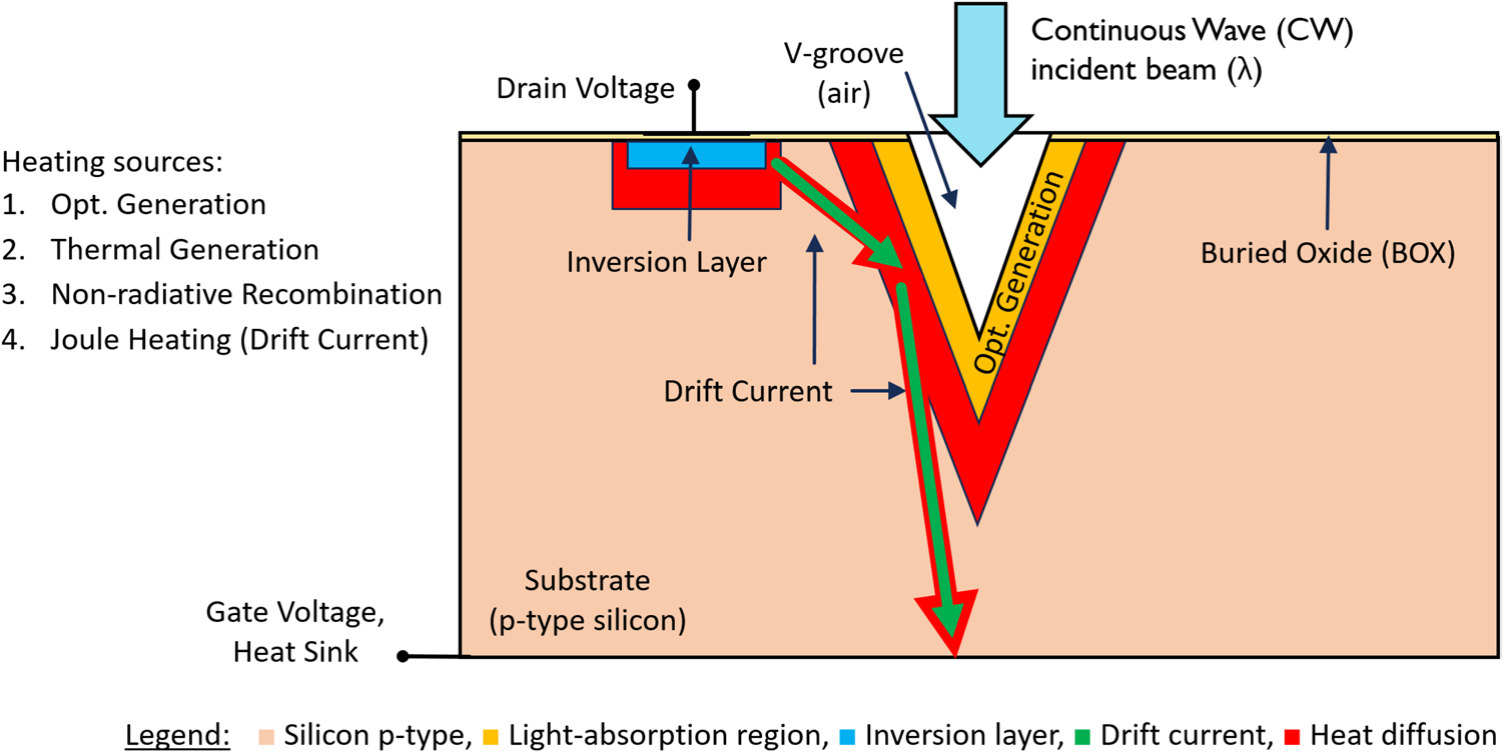
SOIPAM device 2D structure and thermal heating processes.

The device is made of a p-type silicon substrate, a Buried Oxide (BOX) insulator layer above, and an n-type silicon channel at the top. Above the channel, a positive drain voltage is applied, and a negative gate voltage is applied to the substrate. Without illumination, electrons will accumulate under the BOX near the channel, and by electrostatic forces, the channel will be almost complete depletion region. When the device is illuminated, excess charge carriers are created so that more electrons will drift and increase the channel shortage layer, and the canal will be closed. In order to optimize the number of absorbed photons, the illuminated area in the device is designed as a V-groove (Figs. 2, 3), so that more charge carriers will be generated for a given illumination power.

### Between photonic to thermal activation

In the last years, silicon-on-insulator photo-activated modulator (SOIPAM)^13^, and silicon-on-insulator thermo-activated modulator (SOITAM)^14^ have been developed and simulated separately. These devices are silicon-based electro-optical modulators for which the data is electronic, whereas the device control is optic. The benefit of such devices are the ability to smoothly integrate them into existing circuits and the faster response time relative to existed modulators. However, while the previous researches examined the photo-activation separately from the thermal one, this research combines two activation modes into one model and study the relationship between the two processes.

By illuminating the SOIPAM device, two processes are assumed to occur in parallel (Fig. 3). First, a photonic process (absorbed photons generate more pairs of electrons–holes), and second, a thermal process (part of the energy is transformed into heat and entirely lost for current). In other words, there is a loss, and there is a competition between the two processes, while one of them is dominant. The prime purpose of this research is to simulate the two processes inside the device and to develop a complementary analytical model explaining which one of those processes is dominant. This research uses two fundamental working assumptions:

1. Not all the incident illumination process is translated into photons generating pairs of electrons–holes. It may be a second process of thermal generation, creating phonons (vibrations of the atoms and creation of heat). In other words, the loss of energy reduces the speed of operation of the SOIPAM device, and it is not fastest as could be obtained.
2. The entire numerical analysis may involve, at least three combined domains, such as semiconductors, photonics, and heating. The numerical part was written as a Python code.

### Heat study

It is known that in semiconductors, many things depend on the temperature at which the material is. For example, charge carrier concentration, energy gaps, mobility and diffusion coefficients, generation, and recombination physical parameters are influenced by temperature. For example, considering an electro-optical semiconductor-based device, like an avalanche photodiode (APD), performances can deteriorate. Studies have shown^15,16^ that the performance of this type of device decreases as the temperature rises: the total responsiveness (i.e. the amount of current in the diode to the illumination power unit) decreases, the thermal noise increases and as a result, the amount of power required to obtain a good signal to noise ratio (SNR) also increases. Therefore, in investigating the performance of any semiconductor device, it is important to take into account the heat processes generated in the device operation.

The SOIPAM device has three primary heat sources:

1. When the device is illuminated, optical generation is performed. Because silicon is an indirect gap material, some of the photon energy is converted into phonons. Also, the energy of the photon in the visible spectrum is greater than the average energy of an electron–hole pair, so the generation does not utilize all the energy entering the system to create charge carriers. The excess energy is converted into kinetic energy of the charge carriers, which as a result of the collisions also eventually turns into lattice oscillations and heat.
2. Below the channel, there is a large accumulation of electrons, so there is more recombination than a generation. Since silicon is an indirect gap material, it can be assumed that all the energy released in those recombination processes causes the material to heat up.
3. An additional heat source is obtained because of the small currents inside the device that carry the charge from the optical generation area to the deep of the device.

Note that all of the above heat sources not only affect regionally but also diffuse to the inner parts of the device. For draining the heat entering into the system by illumination, a heat sink with a constant temperature is placed at the bottom of the substrate.

The geometry of the device is complex, three-dimensional, and without symmetry, so achieving a complete analytical solution of the equations describing the device is hard. Therefore, the only method left to solve the problem is to use simulation software. Also, solving such a problem can help design similar devices in the future and not just the SOIPAM one. Therefore, the decision was to simplify the device to a more general one-dimensional model and solved it with numerical methods.

**Figure 4:**
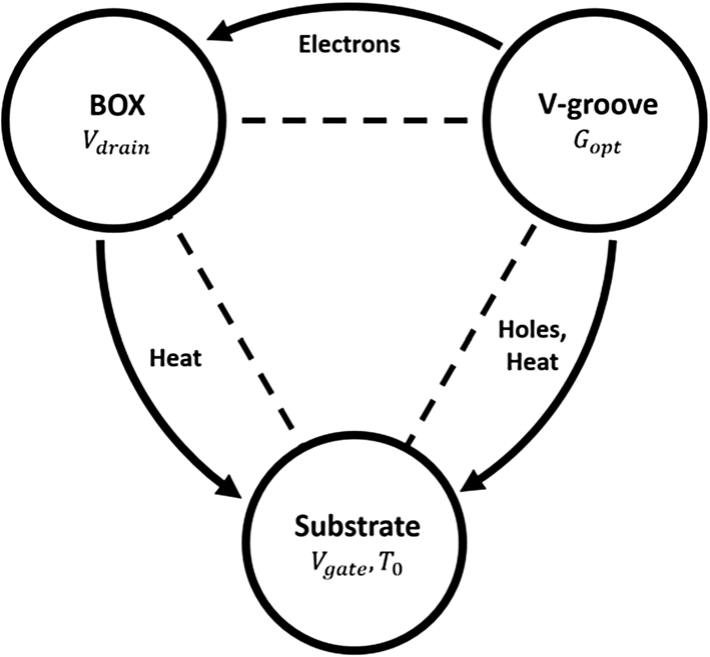
Flow chart of the supposed heat mechanisms and of their interactions.

**Figure 5:**
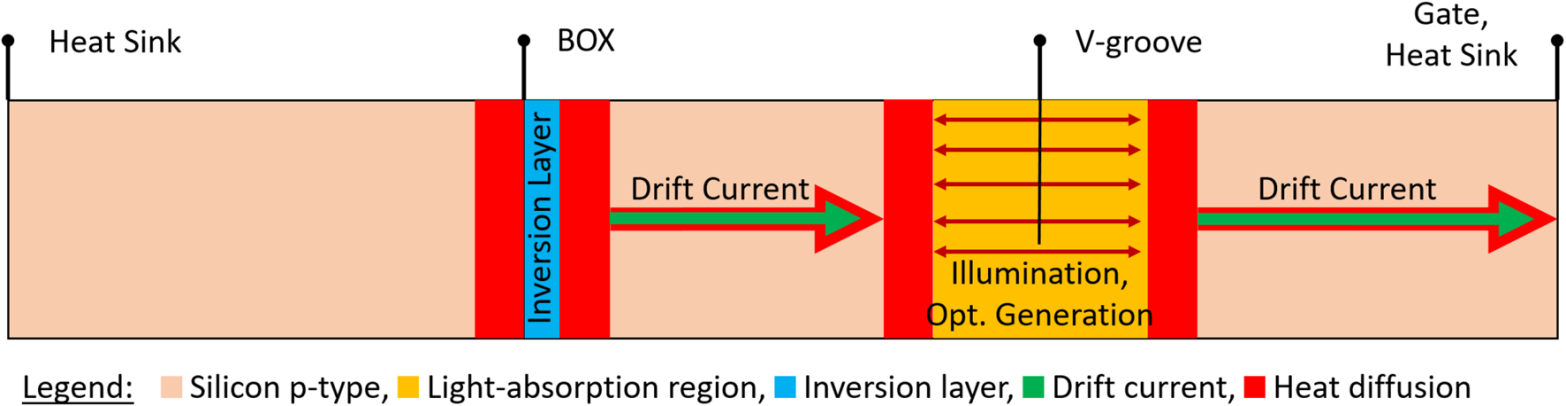
Uni-dimensional model for the heat distribution with diffusion effect (red).

The device has three zones of interest: gate, V-groove, and the BOX below the channel. The interaction between the zones is divided into the semiconductor perspective and the heat one. From the semiconductor perspective, the electrons and holes generated at the V-groove are drifted towards the channel and the gate respectively. From the heat perspective, the heat generated by the optical generation and recombination under the BOX diffuses towards the heat sink. So the following circuit is obtained in Fig. 4:

This circuit can be open and get a one-dimensional structure (Fig. 5), which shares all the characteristics and processes taking place in the original device.

## Simplified 1D analytical model

In order to evaluate the heat distribution versus the photonic generation, it was first needed to create a simplified uni-dimensional model. This 1D model, described in next figures, enables to look at the device from a top view, and to follow the progression of the heat distribution and diffusion effects. Table 1 presents the nomenclature of the parameters used.

**Table 1:** Nomenclature in analytical and numerical developed models.

Sign	Definition	Sign	Definition
$c$	Specific heat	$J_{n} , J_{p}$	Electron and hole current density
$D$	Diffusion coefficient	$n, p$	Electron and hole concentration
$E$	Electrical field	$x_{0}$	V-groove position
$E_{eh}$	Average energy of electron–hole pair	$N_{A}$	Acceptors concentration
$E_{ph}$	Average optical energy for electron–hole pair creation	$N_{D}$	Donors concentration
$P_{0}$	Illumination power per unit area	$R_{net}$	Net recombination rate of charge carriers
$G_{opt}$	Optical generation rate	$T$	Temperature function
$H_{J}$	Joule heat generation rate	$k$	Thermal conductivity
$H_{opt}$	Optical heat generation rate	$\rho$	Mass density
$H_{GR}$	Recombination heat generation rate	$\phi$	Potential
$\alpha$	Absorption coefficient	$\mu$	Mobility coefficient

In order to study the heat-related phenomena, the heat equation in the whole structure needs to be solved. The one-dimensional heat equation in the presence of heat sources is:

1 $$ \rho c\frac{{\partial T\left( {x,t} \right)}}{\partial t} - \frac{\partial }{\partial x}\left( {k \frac{{\partial T\left( {x,t} \right)}}{\partial x}} \right) = H\left( {x, t} \right) $$

where $T$—Temperature function, $k$—Thermal conductivity, $H$—Heat generation rate, $\rho$—Mass density, $c$—Specific heat.

Heat generation rate in the structure is due to optical generation, the excess charge carriers’ recombination and the Joule heating resulting from the current:

2 $$ H = H_{opt} + H_{GR} + H_{J} $$

where

3 $$ H_{opt} = G_{opt} \cdot \left( {E_{ph} - E_{eh} } \right) $$

and

4 $$ H_{GR} = R_{net} \cdot E_{eh} $$

and

5 $$ H_{J} = \left| {J_{n} E} \right| $$

And for which $G_{opt}$—Optical generation rate, $E_{ph}$—Average optical energy for electron–hole pair creation, $E_{eh}$—Average energy of electron–hole pair, $R_{net}$—Net recombination rate of charge carriers in the material, $J_{n}$—Current produced by the electrons, $E$—Electric field.

The optical generation rate is:

6 $$ G_{opt} \left( x \right) = \frac{{\alpha P_{0} }}{{E_{ph} }}e^{{ - \alpha \left| {x - x_{0} } \right|}} $$

where $P_{0}$—Illumination power per unit area, $x_{0}$—V-groove coordinate, $\alpha$—Absorption coefficient.

The boundary condition of this equation is the constant temperature at the both ends of the model. So:

7 $$ T\left( 0 \right) = T\left( {x_{end} } \right) = T_{0} $$

Note that all parameters of the equation are known except for the recombination rate, electrons’ current and the electric field. In order to calculate those parameters, the solution of the semiconductor equations needed to be found.

The basic equations of semiconductors are the equation of continuity, transport, and Laplace (under quasi-static assumption). In one-dimensional:

8 $$ J_{n} = qn\mu_{n} E + qD_{n} \frac{dn}{{dx}} $$

and

9 $$ J_{p} = qp\mu_{p} E - qD_{p} \frac{dp}{{dx}} $$

10 $$ \frac{\partial n}{{\partial t}} = \frac{1}{q}\frac{{\partial J_{n} }}{\partial x} + G_{opt} - R_{net} $$

and

11 $$ \frac{\partial p}{{\partial t}} = - \frac{1}{q}\frac{{\partial J_{p} }}{\partial x} + G_{opt} - R_{net} $$

12 $$ - \frac{{\partial^{2} \phi }}{{\partial x^{2} }} = \frac{\partial E}{{\partial x}} = \frac{\rho }{\epsilon} = \frac{{q\left( {p - n + N_{D} - N_{A} } \right)}}{\epsilon} $$

where $n,p$—Electron and hole concentration respectively, $J$—Current density, $\mu , D$—Mobility and diffusion coefficients respectively, $E, \phi$—Electrical field and potential respectively, $G_{opt}$—Optical generation rate of charge carriers in the material, $R_{net}$—Net trap-assisted recombination rate of charge carriers in the material, $N_{D} , N_{A}$—Donors and acceptors concentration respectively.

The net trap-assisted recombination rate is:

13 $$ R_{net} = \frac{{np - n_{i}^{2} }}{{\tau_{p} \left( {n + n_{t} } \right) + \tau_{n} \left( {p + p_{t} } \right)}} $$

where $n_{i}$—Intrinsic charge carrier concentration, $\tau_{n} , \tau_{p}$—Charge carrier life-time, $n_{t} , p_{t}$—Equilibrium charge carrier concentration.

The boundary condition of this equations is a metal contact at the initial, and the insulator at the BOX with applied voltage $V_{0}$. So:

14 $$ p\left( 0 \right) \approx N_{A} $$

and

15 $$ n\left( 0 \right) \approx \frac{{n_{i}^{2} }}{{N_{A} }} $$

and

16 $$ J_{n} \left( {x_{ins} } \right) = 0 $$

and

17 $$ J_{p} \left( {x_{ins} } \right) = 0 $$

and

18 $$ V\left( 0 \right) = 0 $$

and

19 $$ V\left( {x_{ins} } \right) = V_{0} $$

Note that the parameters $\mu$, $D$ depend on the local temperature of the material, so to solve the above equation, the solution of the heat equation needed to find. In addition, one can see that the equation of heat and the equation of concentration of charge carriers are interdependent. Therefore, those two sets of equations must to be solved at once.

First, the equilibrium state is assumed and the equations solved independently of time:

20 $$ \frac{\partial n}{{\partial t}} = \frac{\partial p}{{\partial t}} = \frac{\partial T}{{\partial t}} = 0 $$

This states describes the structure operation after a long time. Also, the boundary conditions are obtained for a future solution of PDEs (Partial Differential Equations) to describe the full transition between the operating modes of the structure.

## Numerical model

Finite Element Method (FEM) is a numerical method for solving problems of engineering and mathematical physics. The basic procedure starts by modeling the body by dividing it into an equivalent system of many smaller bodies or units, known as finite elements, interconnected at points, known as nodes or nodal points, common to two or more elements or boundary lines. The complete set of elements is known as mesh. The field variable, which is to be solved, is described throughout the body by a set of partial differential equations that are impossible to solve mathematically. Instead, the assumption is that the variable acts through or over each element in a predefined manner. This assumed variation may be, for example, a constant, a linear, a quadratic or a higher order function distribution. The explicit value of the field variable is calculated at each nodal point and then by interpolation is approximated at non-nodal points. Therefore, FEM is used in multi-physics software packages in order to support the design and simulation of physical devices and phenomena. The physical equations are discretized on a mesh. As mentioned above, a Python complex code was developed in order to simulate the different triggers and mechanisms.

**Figure 6:**
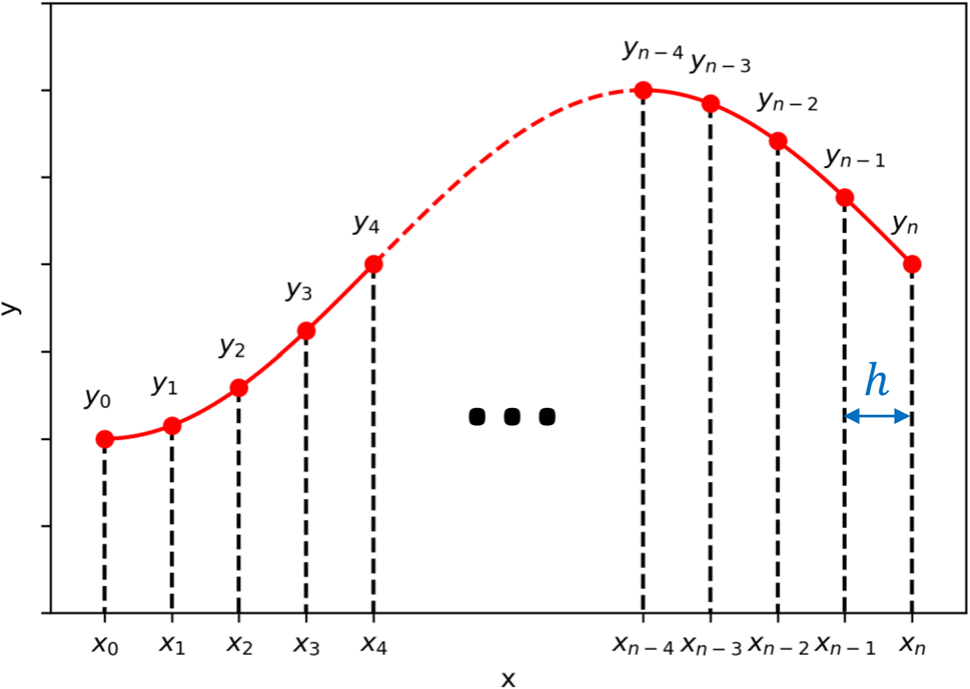
Finite elements method (FEM) discrete domain illustration.

One can see that the resulting differential equations are not simple to solve even in a one-dimensional way. Therefore, it is necessary to use the numerical approach. In this approach, the structure will be divided into discrete cells (Fig. 6) and the differential equations will become recursive:

21 $$ \frac{dy}{{dx}} \approx \frac{{{\Delta }y}}{{{\Delta }x}} = \frac{{y\left[ {x_{i + 1} } \right] - y\left[ {x_{i} } \right]}}{{{\Delta }x}} \approx \frac{{y\left[ {x_{i} } \right] - y\left[ {x_{i - 1} } \right]}}{{{\Delta }x}} $$

The current duo to holes leaving a particular cell $x_{i}$ can now be written:

22 $$ J_{p}^{out} \left[ {x_{i} } \right] = { }qp\left[ {x_{i} } \right]\mu_{p} \left[ {x_{i} } \right]E\left[ {x_{i} } \right] - qD_{p} \left[ {x_{i} } \right]\frac{{p\left[ {x_{i + 1} } \right] - p\left[ {x_{i} } \right]}}{\Delta x} $$

To maintain symmetry around $x_{i}$ in the finite equation, a backward discrete derivative is used and the continuity equation for the holes obtained:

23 $$ \frac{{{\Delta }p}}{{{\Delta }t}} = - \frac{1}{q}\frac{{J_{p} \left[ {x_{i} } \right] - J_{p} \left[ {x_{i - 1} } \right]}}{\Delta x} + G_{opt} \left[ {x_{i} } \right] - R\left[ {x_{i} \left] { + G} \right[x_{i} } \right] $$

By placing, the recursive formula for equilibrium-state obtained:

24 $$ \begin{aligned} & \frac{1}{\Delta x}\left( {p\left[ {x_{i - 1} } \right]\mu_{p} \left[ {x_{i - 1} } \right]E\left[ {x_{i - 1} } \right] - D_{p} \left[ {x_{i - 1} } \right]\frac{{p\left[ {x_{i} } \right] - p\left[ {x_{i - 1} } \right]}}{\Delta x} - p\left[ {x_{i} } \right]\mu_{p} \left[ {x_{i} } \right]E\left[ {x_{i} } \right] + D_{p} \left[ {x_{i} } \right]\frac{{p\left[ {x_{i + 1} } \right] - p\left[ {x_{i} } \right]}}{\Delta x}} \right) \\ & \quad + G_{opt} \left[ {x_{i} } \right] - \left( {R - G} \right)\left[ {x_{i} } \right] = 0 \\ \end{aligned} $$

A recursive equation of $p\left[ {x_{i + 1} } \right]$ was obtained as a function of $p\left[ {x_{i} } \right],{ }p\left[ {x_{i - 1} } \right]$.

Similarly, for the electrons:

25 $$ \begin{aligned} & \frac{1}{\Delta x}\left( {n\left[ {x_{i} } \right]\mu_{n} \left[ {x_{i} } \right]E\left[ {x_{i} } \right] + D_{n} \left[ {x_{i} } \right]\frac{{n\left[ {x_{i + 1} } \right] - n\left[ {x_{i} } \right]}}{\Delta x} - n\left[ {x_{i - 1} } \right]\mu_{n} \left[ {x_{i - 1} } \right]E\left[ {x_{i - 1} } \right] - D_{n} \left[ {x_{i - 1} } \right]\frac{{n\left[ {x_{i} } \right] - n\left[ {x_{i - 1} } \right]}}{\Delta x}} \right) \\ & \quad + G_{opt} \left[ {x_{i} } \right] - \left( {R - G} \right)\left[ {x_{i} } \right] = 0 \\ \end{aligned} $$

where

- The coefficients of mobility and diffusion are obtained as a function of temperature in $x_{i}$.
- The electric field is calculated using the Gauss’s law:
  26 $$ \mathop \int \limits_{s} \vec{E} \cdot d\vec{A} = \frac{{Q_{in} }}{\epsilon} \Rightarrow E\left[ {x_{i} } \right] = \frac{q}{\epsilon}\mathop \sum \limits_{j = 0}^{i} \left( {p\left[ {x_{j} } \right] - N_{A} \left[ {x_{j} } \right] - n\left[ {x_{j} } \right] + N_{D} \left[ {x_{j} } \right]} \right){\Delta }x_{j} $$
- Optical generation rate is:
  27 $$ G_{opt} \left[ {x_{i} } \right] = \alpha \frac{{P_{opt} }}{{E_{ph} }} = \frac{{\alpha P_{0} }}{{E_{ph} }}e^{{ - \alpha \left| {x_{i} - x_{o} } \right|}} $$
  where $P_{0}$—Illumination, $x_{0}$—V-groove coordinate, $\alpha$—Absorption coefficient.
- Thermal recombination-generation rate:
  28 $$ R - G = \frac{{\hat{p}}}{{\tau_{n} }} + \frac{{\hat{n}}}{{\tau_{p} }} = \frac{{p - n_{i} e^{{ - \frac{q\phi }{{k_{B} T}}}} }}{{\tau_{n} }} + \frac{{n - n_{i} e^{{\frac{q\phi }{{k_{B} T}}}} }}{{\tau_{p} }} $$
  where $\hat{n},{ }\hat{p}$—Excess electrons and holes concentrations respectively, $n_{i}$—Intrinsic electrons and holes concentrations, $\tau_{n} ,{ }\tau_{p}$—Electrons and holes life time, $\phi$—Electrical potential obtained by Poisson law:
  29 $$ \frac{{\partial^{2} \phi }}{{\partial x^{2} }} = - \frac{\rho }{\epsilon} $$

Note that each recursion function make use of an electrical field and potential that depends on both electrons and holes concentration. So it is impossible to solve each equation separately, but it has to be done together step by step.

To obtain a complete numerical solution, two boundary condition are required for each equation:

1. At the gate, the voltage is zero, and there is no excess charge carrier, so the charge carrier concentration for p-type silicon is given by:
  30 $$ p\left[ {x_{0} } \right] \approx N_{A} $$
  and
  31 $$ n\left[ {x_{0} } \right] = \frac{{n_{i}^{2} }}{{N_{A} }} $$
2. There is no current through the insulator layer ($J_{p}^{out} \left[ {x_{end} } \right] = 0$), so the semi-conductor equations must also comply with the following condition:
  32 $$ \frac{1}{q}\frac{{J_{p} \left[ {x_{end - 1} } \right]}}{\Delta x} + G_{opt} \left[ {x_{end} } \right] - R\left[ {x_{end} } \right] + G\left[ {x_{end} } \right] = 0 $$

From here we get:

33 $$ \begin{aligned} & \frac{1}{\Delta x}\left( {p\left[ {x_{end - 1} } \right]\mu_{p} \left[ {x_{end - 1} } \right]E\left[ {x_{end - 1} } \right] - D_{p} \left[ {x_{end - 1} } \right]\frac{{p\left[ {x_{end} } \right] - p\left[ {x_{end - 1} } \right]}}{\Delta x}} \right) \\ & \quad + G_{opt} \left[ {x_{end} } \right] - \left( {R - G} \right)\left[ {x_{end} } \right] = 0 \\ \end{aligned} $$

Similarly, for the electrons:

34 $$ \begin{aligned} & \frac{1}{\Delta x}\left( { - n\left[ {x_{end - 1} } \right]\mu_{n} \left[ {x_{end - 1} } \right]E\left[ {x_{end - 1} } \right] - D_{n} \left[ {x_{end - 1} } \right]\frac{{n\left[ {x_{end} } \right] - n\left[ {x_{end - 1} } \right]}}{\Delta x}} \right) \\ & \quad + G_{opt} \left[ {x_{end} } \right] - \left( {R - G} \right)\left[ {x_{end} } \right] = 0 \\ \end{aligned} $$

Note that $x_{end}$ refers to the end of the semiconductor model, at the insulator layer ($x_{ins}$). The numerical solution must meet these two boundary conditions. Note that there is another condition concerning the two equations, and that is that the voltage difference applied between the ends of the structure must correspond to the voltage given by the Poisson equation:

35 $$ \frac{{\partial^{2} \phi }}{{\partial x^{2} }} = - \frac{\partial E}{{\partial x}} = - \frac{\rho }{\epsilon} \Rightarrow \phi \left( x \right) - \phi \left( 0 \right) = - \mathop \int \limits_{0}^{x} E dx = - \frac{1}{\epsilon}\mathop \int \limits_{0}^{x} \mathop \int \limits_{0}^{{x^{\prime}}} \rho dx^{\prime\prime}dx^{\prime} $$

And in a discrete form in p-type silicon:

36 $$ V = - \frac{{\text{q}}}{\epsilon}\mathop \sum \limits_{i = 0}^{end} \mathop \sum \limits_{j = 0}^{i} \left( {p\left[ j \right] - n\left[ j \right] - N_{A} \left[ j \right]} \right)\left( {{\Delta }x} \right)^{2} $$

After semiconductor problem solution, a heat-equation can be solved:

37 $$ k \cdot \frac{{T\left[ {x_{i + 1} } \right] - 2T\left[ {x_{i} } \right] + T\left[ {x_{i - 1} } \right]}}{{{\Delta }x^{2} }} + G_{opt} \left[ {x_{i} } \right]\left( {E_{ph} - E_{eh} } \right) + \left( {R - G} \right)\left[ {x_{i} } \right]E_{eh} = 0 $$

Its recursive equation of $T\left[ {x_{i + 1} } \right]$ as a function of $T\left[ {x_{i} } \right],{ }T\left[ {x_{i - 1} } \right]$. In this equation the assumption is that the heat capacity and heat conductive are approximately constants, so they can be dropped out from the derivative. To obtain a complete numerical solution, two boundary condition are required. At both ends of the structure, there is a heat sink with a constant temperature $T_{0}$, so the boundary conditions are:

38 $$ T\left[ {x_{0} } \right] = T\left[ {x_{end} } \right] = T_{0} $$

## Analytical approximated solution: free carriers associated with solving localized heating

We will perform the analysis for the p-type region of the semi-conductor. The equations we work with are Eqs. (9), (11) and (12). By substituting the equations above into each other results with:

39 $$ - p\mu_{p} \frac{{p - N_{A} }}{\epsilon} + D_{p} \frac{{\partial^{2} p}}{{\partial x^{2} }} = \frac{\partial p}{{\partial t}} - \left( {G_{p} \left( t \right) - R_{p} \left( t \right)} \right) $$

We will assume that:

40 $$ p = N_{A} + {\Delta }p = N_{A} \left( {1 + \frac{{{\Delta }p}}{{N_{A} }}} \right) $$

and that

41 $$ {\Delta }p \ll N_{A} $$

Thus,

42 $$ p^{2} \approx N_{A}^{2} \left( {1 + 2\frac{{{\Delta }p}}{{N_{A} }}} \right) = N_{A}^{2} + 2N_{A} {\Delta }p $$

Therefore, Eq. (39) will become as follows:

43 $$ - p\mu_{p} \frac{{{\Delta }p}}{\epsilon} + D_{p} \frac{{\partial^{2} {\Delta }p}}{{\partial x^{2} }} = \frac{{\partial {\Delta }p}}{\partial t} - \left( {G_{p} \left( t \right) - R_{p} \left( t \right)} \right) $$

Following our former assumption, we will also assume that:

44 $$ {\Delta }p^{2} \ll N_{A} {\Delta }p $$

And notating:

45 $$ F\left( t \right) \equiv - \left( {G_{p} \left( t \right) - R_{p} \left( t \right)} \right) $$

Resulting with:

46 $$ - N_{A} \mu_{p} \frac{{{\Delta }p}}{\epsilon} + D_{p} \frac{{\partial^{2} {\Delta }p}}{{\partial x^{2} }} = \frac{{\partial {\Delta }p}}{\partial t} + F\left( t \right) $$

We will assume separation of variables:

47 $$ {\Delta }p = {\Delta }p\left( {x,t} \right) = {\Delta }p_{x} \left( x \right){\Delta }p_{t} \left( t \right) $$

Leading to:

48 $$ - N_{A} \mu_{p} \frac{{{\Delta }p_{x} {\Delta }p_{t} }}{\epsilon} + D_{p} {\Delta }p_{t} \frac{{\partial^{2} {\Delta }p_{x} }}{{\partial x^{2} }} = {\Delta }p_{x} \frac{{\partial {\Delta }p_{t} }}{\partial t} + F\left( t \right) $$

Yielding:

49 $$ - N_{A} \frac{{\mu_{p} }}{\epsilon} + \frac{{D_{p} }}{{{\Delta }p_{x} }}\left( {\frac{{\partial^{2} {\Delta }p_{x} }}{{\partial x^{2} }}} \right) = \frac{1}{{{\Delta }p_{t} }}\left( {\frac{{\partial {\Delta }p_{t} }}{\partial t}} \right) + \frac{F\left( t \right)}{{{\Delta }p_{x} {\Delta }p_{t} }} $$

We will assume that the external illumination function equals to a series of *rect* pulses as happens when functions are represented using Haar transform (Rademacher system consisting of sums of Haar functions):

50 $$ F\left( t \right) = \mathop \sum \limits_{n} f_{n} rect\left( {\frac{{t - \delta t_{n} }}{{{\Delta }t_{n} }}} \right) $$

For the sake of linearity (we have linear differential equations), we will solve it for a single *rect* series:

51 $$ F\left( t \right) = f\mathop \sum \limits_{n} rect\left( {\frac{t - n\delta t}{{{\Delta t}}}} \right) $$

For the case of the temporal regions where the *rect* is zero, we have the differential equation of:

52 $$ - N_{A} \frac{{\mu_{p} }}{\epsilon} + \frac{{D_{p} }}{{{\Delta }p_{x} }}\left( {\frac{{\partial^{2} {\Delta }p_{x} }}{{\partial x^{2} }}} \right) = \frac{1}{{{\Delta }p_{t} }}\left( {\frac{{\partial {\Delta }p_{t} }}{\partial t}} \right) = const = F_{0} $$

And we have solvable first and second order regular (instead of partial) differential linear equation for the time and the space axes:

53 $$ \frac{{\partial {\Delta }p_{t} }}{\partial t} - {\Delta }p_{t} F_{0} = 0 $$

and

54 $$ D_{p} \left( {\frac{{\partial^{2} {\Delta }p_{x} }}{{\partial x^{2} }}} \right) - {\Delta }p_{x} \left( {N_{A} \frac{{\mu_{p} }}{\epsilon} + F_{0} } \right) = 0 $$

The solutions for those equations are:

55 $$ {\Delta }p_{t} \left( t \right) = {\Delta }p_{t} \left( 0 \right){\text{exp}}\left( {F_{0} t} \right) $$

and

56 $$ {\Delta }p_{x} \left( x \right) = A_{1} \exp \left( {\lambda_{0} x} \right) + A_{2} {\text{exp}}\left( { - \lambda_{0} x} \right) $$

where

57 $$ \lambda_{0} = \sqrt {\frac{{\left( {N_{A} \frac{{\mu_{p} }}{\epsilon} + F_{0} } \right)}}{{D_{p} }}} A_{1} = \frac{{{\Delta }p_{x} \left( L \right)\exp \left( {\lambda_{0} L} \right) - {\Delta }p_{x} \left( 0 \right)}}{{exp\left( {2\lambda_{0} L} \right) - 1}} A_{2} = {\Delta }p_{x} \left( 0 \right) - A_{1} $$

while the size of the p type region is *L.*

For the case of the temporal regions where the *rect* is not zero but rather equals to the value of *f*, we have the differential equation of:

58 $$ - N_{A} \frac{{\mu_{p} }}{\epsilon}{\Delta }p_{x} + D_{p} \left( {\frac{{\partial^{2} {\Delta }p_{x} }}{{\partial x^{2} }}} \right) = \frac{{{\Delta }p_{x} }}{{{\Delta }p_{t} }}\left( {\frac{{\partial {\Delta }p_{t} }}{\partial t}} \right) + \frac{f}{{{\Delta }p_{t} }} $$

We will derive both wings according to the space axis and receive:

59 $$ - N_{A} \frac{{\mu_{p} }}{\epsilon}\left( {\frac{{\partial {\Delta }p_{x} }}{\partial x}} \right) + D_{p} \left( {\frac{{\partial^{3} {\Delta }p_{x} }}{{\partial x^{3} }}} \right) = \frac{1}{{{\Delta }p_{t} }}\left( {\frac{{\partial {\Delta }p_{t} }}{\partial t}} \right)\left( {\frac{{\partial {\Delta }p_{x} }}{\partial x}} \right) $$

which yields to:

60 $$ - N_{A} \frac{{\mu_{p} }}{\epsilon} + D_{p} {\raise0.7ex\hbox{${\left( {\frac{{\partial^{3} {\Delta }p_{x} }}{{\partial x^{3} }}} \right)}$} \!\mathord{\left/ {\vphantom {{\left( {\frac{{\partial^{3} {\Delta }p_{x} }}{{\partial x^{3} }}} \right)} {\left( {\frac{{\partial {\Delta }p_{x} }}{\partial x}} \right)}}}\right.\kern-\nulldelimiterspace} \!\lower0.7ex\hbox{${\left( {\frac{{\partial {\Delta }p_{x} }}{\partial x}} \right)}$}} = \frac{1}{{{\Delta }p_{t} }}\left( {\frac{{\partial {\Delta }p_{t} }}{\partial t}} \right) = const = F_{1} $$

And we now have solvable first and third order regular (instead of partial) differential linear equation on time and on space axes:

61 $$ \frac{{\partial {\Delta }p_{t} }}{\partial t} - {\Delta }p_{t} F_{1} = 0 $$

and

62 $$ D_{p} \left( {\frac{{\partial^{3} {\Delta }p_{x} }}{{\partial x^{3} }}} \right) - \left( {\frac{{\partial {\Delta }p_{x} }}{\partial x}} \right)\left( {N_{A} \frac{{\mu_{p} }}{\epsilon} + F_{1} } \right) = 0 $$

with solution of:

63 $$ {\Delta }p_{t} \left( t \right) = {\Delta }p_{t} \left( 0 \right){\text{exp}}\left( {F_{1} t} \right) $$

and

64 $$ {\Delta }p_{x} \left( x \right) = \frac{{B_{1} }}{{\lambda_{1} }}\exp \left( {\lambda_{1} x} \right) - \frac{{B_{2} }}{{\lambda_{1} }}\exp \left( { - \lambda_{1} x} \right) + B_{3} $$

where

65 $$ \lambda_{1} = \sqrt {\frac{{\left( {N_{A} \frac{{\mu_{p} }}{\epsilon} + F_{1} } \right)}}{{D_{p} }}} $$

66 $$ B_{1} = \frac{{\frac{{\partial {\Delta }p_{x} }}{\partial x}\left( L \right)\exp \left( {\lambda_{1} L} \right) - \frac{{\partial {\Delta }p_{x} }}{\partial x}\left( 0 \right)}}{{exp\left( {2\lambda_{1} L} \right) - 1}} $$

67 $$ B_{2} = \frac{{\partial {\Delta }p_{x} }}{\partial x}\left( 0 \right) - B_{1} $$

68 $$ B_{3} = {\Delta }p_{x} \left( 0 \right) - \frac{{B_{1} }}{{\lambda_{1} }} + - \frac{{B_{2} }}{{\lambda_{1} }} $$

The bottom line of this mathematical analysis is that it provides an analytical solution to a complicated partial differential equation describing the temporal-spatial distribution of free carriers, which are directly associated with solving the localized heating of the proposed device as appears in the next section.

## Numerical results

For the equation numerical solution, firstly, the equations must be simplified to a first-order ordinary-differential-equation (ODE) system. Secondly, since the concentration of the charge carrier involves values in a vast range, it must be normalized to smaller values. Therefore, the following definitions were used:

69 $$ N \equiv \ln \left( {\frac{n}{{n_{i} }}} \right) $$

and

70 $$ P \equiv \ln \left( {\frac{p}{{n_{i} }}} \right) $$

By determinate appropriate variables for the solution such as:

- Log of normalized charge carrier concentration ($N, P$).
- Charge carrier current ($J_{n} , J_{p}$).
- Electrical field ($$E$$).
- Electrical potential ($$V$$).
- Heat flow per unit area (using unidimensional Fourier law, $h = - k\frac{dT}{{dx}}$).
- Temperature ($$T$$).

And using Einstein relation, one can obtain the following first-order ODE system:

71 $$ \frac{dN}{{dx}} = \frac{{J_{n} }}{{qD_{n} n_{i} e^{N} }} - \frac{qE}{{kT}} $$

72 $$ \frac{dP}{{dx}} = - \left( {\frac{{J_{p} }}{{qD_{p} n_{i} e^{P} }} - \frac{qE}{{kT}}} \right) $$

73 $$ \frac{{dJ_{n} }}{dx} = q\left( {R_{net} - G_{opt} } \right) $$

74 $$ \frac{{dJ_{p} }}{dx} = - q(R_{net} - G_{opt} ) $$

75 $$ \frac{dh}{{dx}} = H $$

76 $$ \frac{dT}{{dx}} = - \frac{h}{k} $$

For the equation solution, the model parameters in Table 2 are used. Those parameters are chosen based on the real SOIPAM device typically values.

**Table 2:** Model’s parameters for the numerical solution.

Variable	Definition	Value
d_1_	Gate-groove distance	$10\;\upmu {\text{m}}$
d_2_	Groove-insulator distance	$10 \;\upmu {\text{m}}$
d_3_	Insulator thickness	$150\;{\text{nm}}$
d_4_	Insulator-gate distance	$10\;\upmu {\text{m}}$
N_a_	Acceptors concentration	$10^{15} \;{\text{cm}}^{ - 3}$
V_0_	Insulator voltage	$1\;{\text{V}}$
Power	Illumination power	$\sim 1\;{\text{mW}}$
A_I_	Illumination area	$\sim 4\;{\text{mm}}^{2}$
A_V_	V-groove illumination entrance area	$\sim 84\;\upmu {\text{m}}^{2}$
λ	Illumination wavelength	$550\;{\text{nm}}$
T_0_	Heat-sink temperature	$300\;{\text{K}}$

Firstly, the approximate solution of the heat equation will be found, for the getting sense of how much the model could be heated. After that, a completely numerical solution using python will be shown.

### Approximate analytical solution

Using the heat equation and some assumption, the approximated analytical solution can be achieved. Let assume that heat flow $h_{0}$ entering from the one side of the model (instead of volume heat generation), and the thermal conductivity is constant. So:

77 $$ \frac{dh}{{dx}} = 0 \Rightarrow h = h_{0} = {\text{const}} $$

Then one can obtain:

78 $$ \frac{dT}{{dx}} = - \frac{{h_{0} }}{k} = {\text{const}} \Rightarrow T = - \frac{h}{k}x + T\left( 0 \right) $$

If a heat-sink is defined at the end of the model so $T\left( L \right) = T_{0}$, then the difference in temperature along the model will be:

79 $$ {\Delta }T\left( x \right) = T\left( x \right) - T_{0} = \frac{h}{k}\left( {L - x} \right) $$

So the temperature heating in the model is dependent linearly in the heat-flow entering the model and the heat-sink distance from the entrance point (the thermal conductivity is dependent on the material, and couldn’t be changed, for Silicon $k \approx 1.3\frac{{\text{W}}}{{{\text{cm}}\;{\text{K}}}}$).

### Python scripts for semiconductor equation solve

The Python programing language script receive the numerical solution of the model. This script uses the Scipy (scientific-python) library to solve the system of ODE mentioned above with boundary conditions. Since the equations are not simple to solve, a continuous-solution method is applied: instead of solving the equation for the final condition immediately, the solution for the simpler case was found, and it serves as an initial guess to the next step of the solution. In such a way, the complete solution is achieved after several iterations.

The physical properties of the model (such as semiconductor and heat properties), were achieved from some sources, and stored in a different file for script use. Among other used properties, one can note the charge carrier mobility versus temperature and doping level^17^, the absorption length versus wavelength^18^, the electron–hole pair creation energy versus wavelength^19^, the silicon band structure and carrier concentration properties^20^, the electrical properties of silicon^21^, and the thermal properties of silicon^22^.

### Numerical solution

#### No-illumination

**Figure 7:**
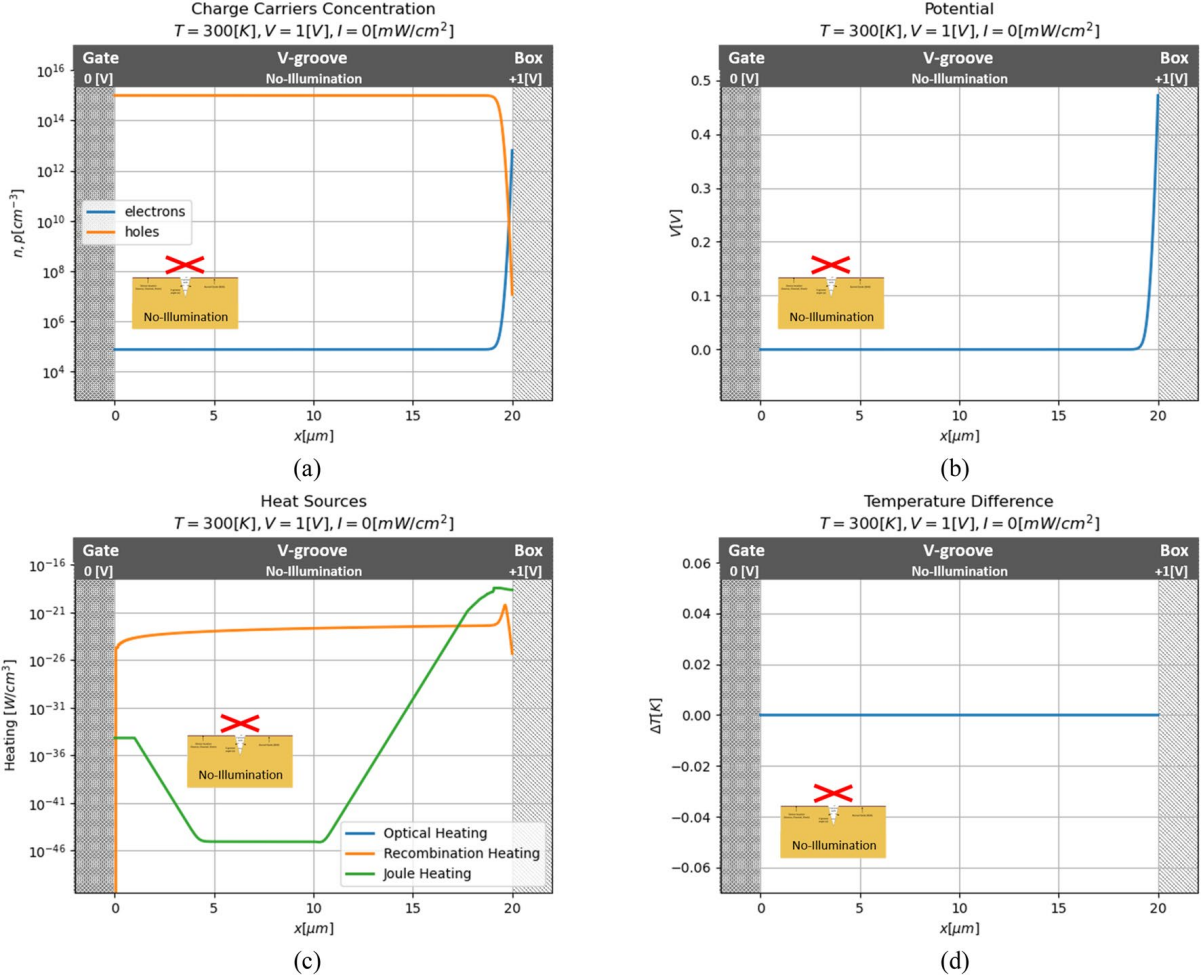
Series of numerical results for no-illumination case. (**a**) Charge carriers concentration; (**b**) potential; (**c**) heat sources; (**d**) temperature distribution.

Firstly, the solution of the device’s ON mode will be presented. In this mode, no illumination is applied to the device, and therefore its acts as a regular MOS capacitor. i.e., the charge carrier concentration uniformly distributed in the material, except the insulator region, there is a strong increase in the electrons' concentration and a decrease in the hole's one. In such a way, all potential is applied on the one small region of the material near the insulator layer. Those results are shown in Fig. 7.

For this state, extremely small heat sources are presented. As it is mentioned above (Eq. 5), the Joule heating depends on the electron current, and the electrical field. But, while the most electrical field presence near the insulator layer, the current is significantly larger only near the gate. So the Joule heating increases at the ends of the model and decreases at the middle. In addition, the recombination rate increases near the insulator and decreases again immediately. This phenomenon can be explained by the fact that recombination rate is dependent on the presence of both types of charge carriers, so only while the concentration of electrons increases and the concentration of the hole didn’t decrease to a very small value, the heating occurs. Therefore, is no temperature increasing occurs, and the model present at the constants temperature defined by the heat-sinks.

#### Simple illumination

**Figure 8:**
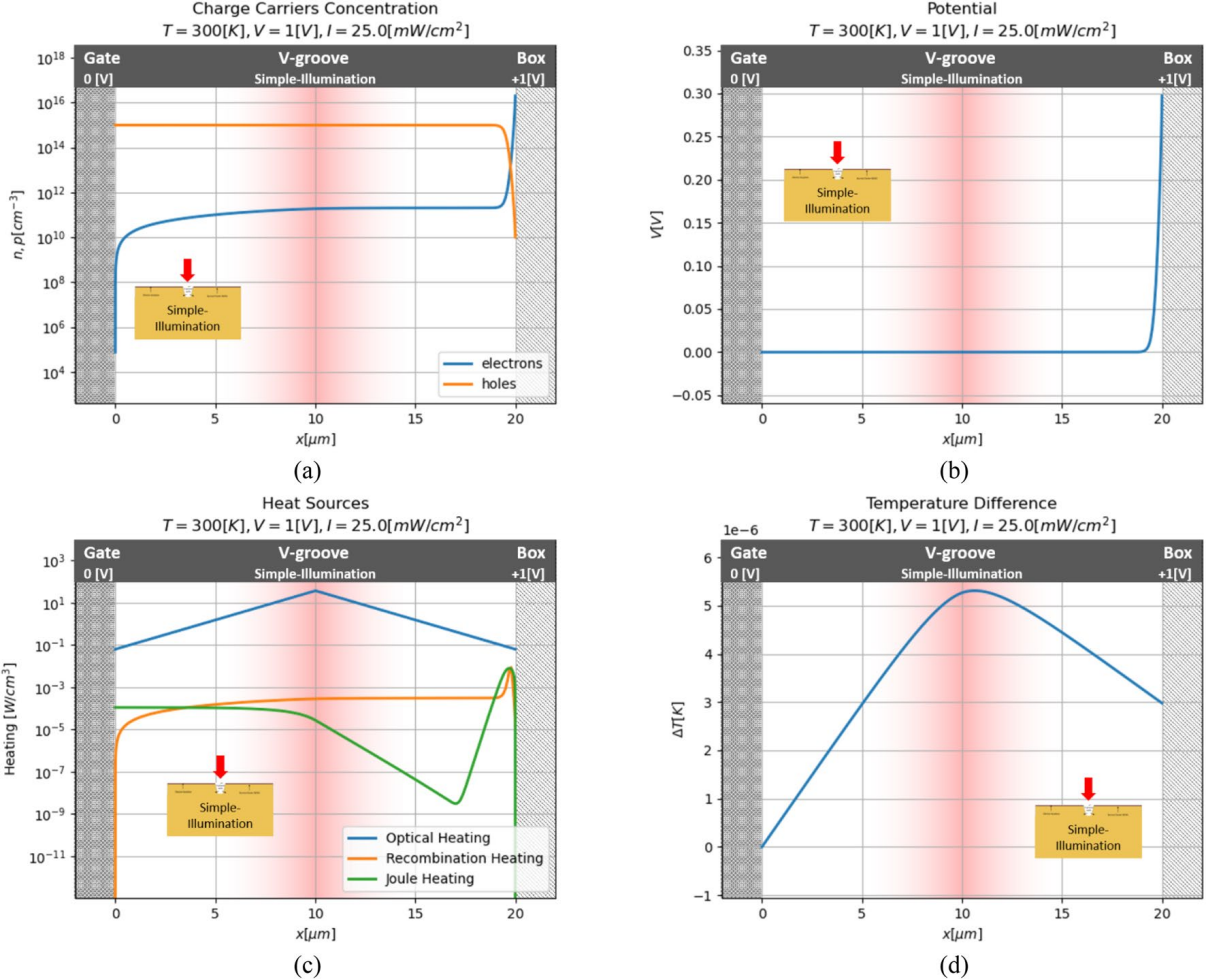
Series of numerical results for simple case. (**a**) Charge carriers concentration; (**b**) potential; (**c**) heat sources; (**d**) temperature distribution.

Now, the solution of the device’s OFF mode is presented for a simple condition. In this mode, laser illumination is applied to the model without light concentration, so the illumination power per unit area is defined by the laser beam size only. Using the parameters of the model defined above, the illumination power per unit area of $25\;{\text{mW/cm}}^{2}$ is obtained. The result for this condition is presented in Fig. 8.

Now, the electrons' concentration extremely increases duo to the optical carrier generation (the hole concentration almost didn't change because the optical generation is smaller from the acceptors' concentration in the material). By that, more electrons are concentrated now near the insulator, and the channel of the device will be close. In this condition, the heat sources are more significant and have more influence on the temperature of the model. But, since the illumination is not concentrated to the area of the v-groove, those heating sources aren't sufficient enough to increase the temperature more than on the few micro-Kelvin.

#### Concentrated illumination

**Figure 9:**
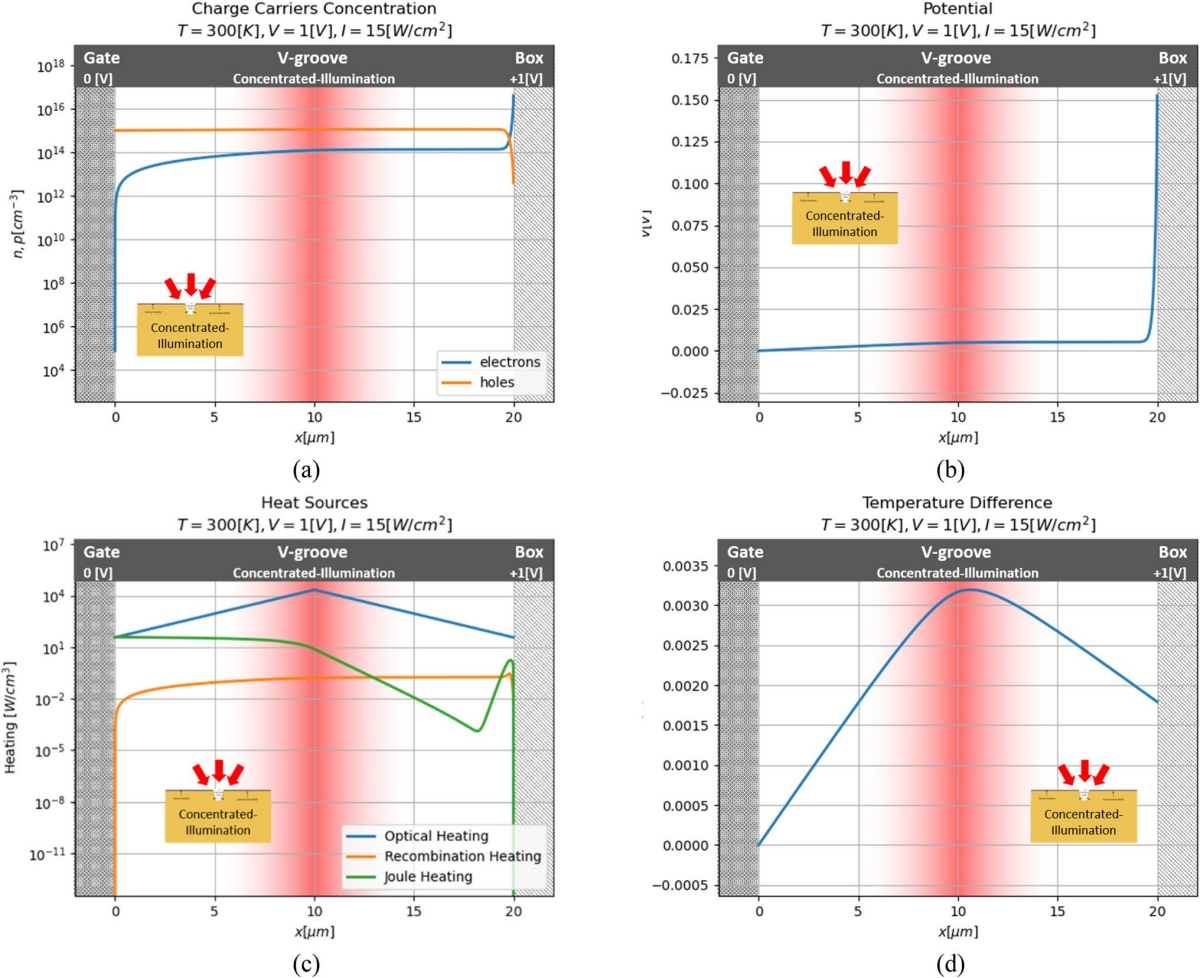
Series of numerical results for concentrated illumination case. (**a**) Charge carriers concentration; (**b**) potential; (**c**) heat sources; (**d**) temperature distribution.

Since the device's size is in order of several micrometers, illumination without beam concentration isn't efficient. For better performance, the illumination needed to be concentrated into a small beam pointed to the v-groove. Under this condition, the heating process in the model will be more significant than the result showed above. In this section, the illumination is concentrated 600 times than the regular laser beam. The results are shown in the figure below (Fig. 9).

Now, one can see that the illumination effect is much stronger and, the electrons' concentration near the insulator is significantly larger. But, the heat sources also became much powerful than previous. Note, that meanwhile, the Joule heating in the previous simulation was negligible relative to the optical one, now this heating source is larger and at some point contributes no less than the optical heating. Those growth heating sources cause a much more significant increase in the temperature.

#### Real conditions

**Figure 10:**
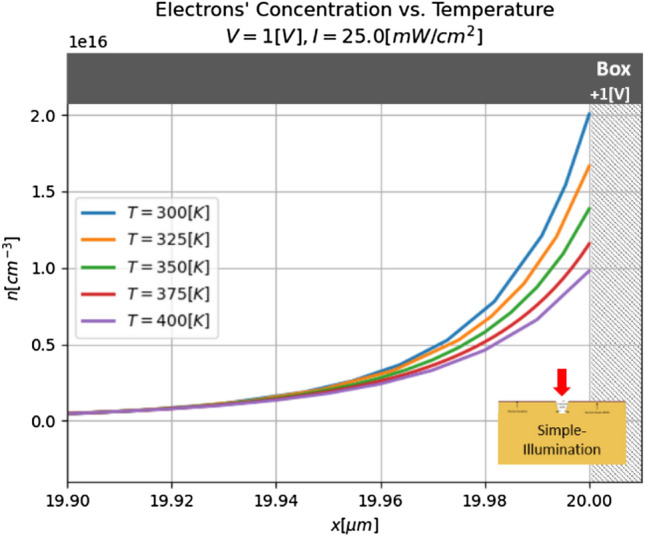
Electrons’ concentration near the insulator for different temperatures and no-concentrated illumination.

Although the temperature increasing shown above are relatively small, in the real-world case, all power of the illumination should enter the v-groove. In such a way, and using the parameters of the model defined above, the illumination power per unit area of 1.2 [kW/cm^2^] is obtained. For such a power, the temperature increase will be higher than the result presented above. In addition, in the real world, the heat sink is not located so close to the device, so one can expect that the temperature will be even higher than those that the simplified model predicts. These temperature increases affected the efficiency of the device, so for the parameters, the electrons' concentration near the insulator is smaller for the higher temperatures (see Fig. 10). This phenomenon causes to increase in illumination power to get the same effect, as it was not warmed.

### Identifying optimal parameters

**Figure 11:**
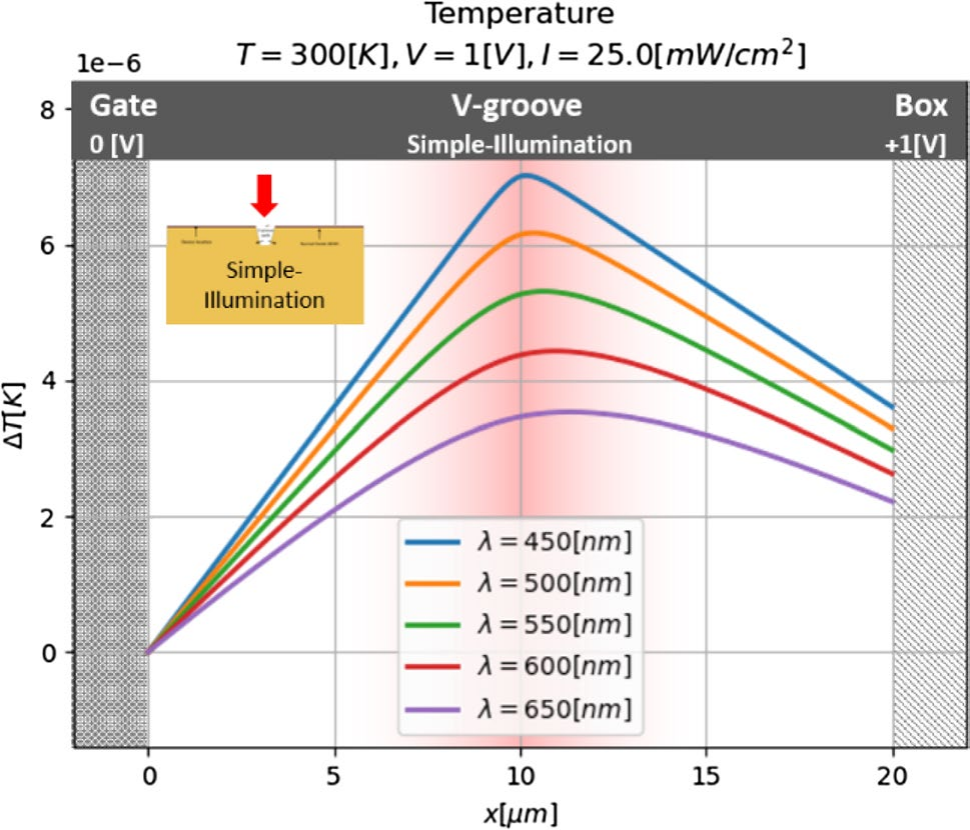
Temperature of the model for several wavelengths and no-concentrated illumination.

Much more simulation runs will now be performed in order to identify in which conditions one of the two processes, photonic or thermal, is dominant, and what should be the optimal wavelength. One can see (Fig. 11), that the longer the wavelength, the smaller the temperature. This result can be explained by the fact that how light is closer to the cut-off wavelength, then less energy remains in the optical generation process, and therefore less power goes to heating, and the model remains not such heated.

## Picosecond pulsed laser versus CW laser

### Proposed solution: short pulses instead of CW

**Figure 12:**
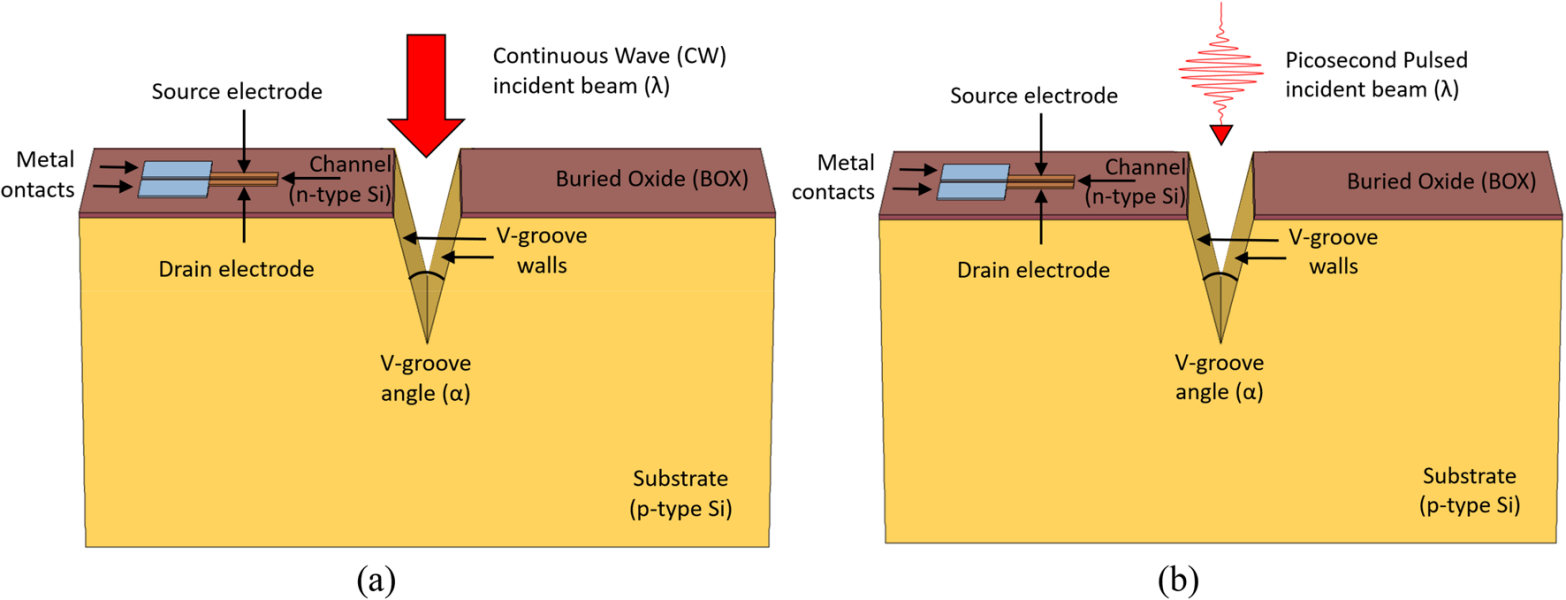
Illumination incident beam. (**a**) Continuous wave laser; (**b**) picosecond pulsed laser.

The last two decades, researchers investigated the usage and the limits of very short pulses (i.e. femtosecond, picosecond, and nanosecond lasers)^23^. The analyses concluded that, for an applied range of laser pulse duration, there is a barrier of 1 ps, and two regimes can be distinguished: Below and above the 1 ps value. In spite of the advantage of relative low-heating effects while using pulses, heat accumulation issues were studied during pulsed laser materials processing^24^. Some research investigated some numerical models of the heat transfer, for specific material removal, which is not our case, in using picosecond laser^25^. As per our analyses above, it clearly appears that a significant part of the illumination beam is converted in several unnecessary heating effects. Looking at the setup, a continuous wave (CW) of laser incident beam is projected on the photo-activated modulator (Fig. 12a). In order to prevent undesirable heating phenomena, it is now proposed to illuminate the sample with illuminating picosecond pulses (Fig. 12b), so no energy will be wasted in heat. When comparing illuminating pulses beam to CW regular one, one can observe how the thermal efficiency will change accordingly. In other words, assuming a pulse of “1”, itself made of series of very short pulses, sharing a magnitude order of 1 picosecond, can be the solution. The length of such pulses, determining the optimization of the incident beam, is itself a full subject of research.

### Laser picosecond pulses analytical model

The thermodynamic equation is:

80 $$ \rho AP\left( t \right) = A\varepsilon \sigma T\left( t \right)^{4} + \alpha \left( {T\left( t \right) - T_{0} } \right) + C\frac{dT\left( t \right)}{{dt}} $$

where $\rho$ is the absorption coefficient of the laser light in the illuminated device. *A* is the area of the device, and *P(t)* is the power density of the illuminating laser’s pulses.

In case of pulsed laser we have:

81 $$ P\left( t \right) = P_{0} \mathop \sum \limits_{n} rect\left( {\frac{t - n\Delta t}{{\delta t}}} \right) $$

While $\Delta t$ is the temporal periodicity of the pulses and $\delta t$ is the temporal length of each pulse.

In the right wing, we have three terms. The first is the Stefan–Boltzmann law where $\varepsilon$ is the emissivity of the device, $\sigma$ is the Stefan–Boltzmann constant ($\sigma$ = 5.67 × 10^−8^ [W/m^2^/°K^4^]), and *T*(*t*) is the change of the temperature of the device versus time. $\alpha$ is the coefficient describing the heat conduction and convection. $T_{0}$ is the ambient temperature and C is the heat capacity. Approximately:

82 $$ C \approx mC_{p} $$

where *m* is the mass of the device and $C_{p}$ is the specific heat capacity.

One needs to remember that $T_{0} \approx 300^{{\circ}}{\text{K}}$, we may assume that:

83 $$ T\left( t \right) = T_{0} + \Delta T\left( t \right) = T_{0} \left( {1 + \frac{\Delta T\left( t \right)}{{T_{0} }}} \right) $$

where $${\raise0.7ex\hbox{${\Delta T\left( t \right)}$} \!\mathord{\left/ {\vphantom {{\Delta T\left( t \right)} {T_{0} }}}\right.\kern-\nulldelimiterspace} \!\lower0.7ex\hbox{${T_{0} }$}} \ll 1$$ and therefore after taking only the first term of the Taylor expansion one gets:

84 $$ A\varepsilon \sigma T\left( t \right)^{4} = A\varepsilon \sigma T_{0}^{4} \left( {1 + \frac{\Delta T\left( t \right)}{{T_{0} }}} \right)^{4} \approx A\varepsilon \sigma T_{0}^{4} \left( {1 + \frac{4\Delta T\left( t \right)}{{T_{0} }}} \right) $$

and the differential equitation becomes:

85 $$ \rho AP\left( t \right) - A\varepsilon \sigma T_{0}^{4} = \left( {4A\varepsilon \sigma T_{0}^{3} + \alpha } \right)\Delta T\left( t \right) + C\frac{d\Delta T\left( t \right)}{{dt}} $$

which can be written as:

86 $$ \mathop \int \limits_{{t_{s} }}^{{t_{f} }} dt = \mathop \int \limits_{{\Delta T_{s} }}^{{\Delta T_{f} }} \frac{C}{{\rho AP\left( t \right) - A\varepsilon \sigma T_{0}^{4} - \left( {4A\varepsilon \sigma T_{0}^{3} + \alpha } \right)\Delta T\left( t \right)}}d\Delta T\left( t \right) $$

where $\Delta T_{s}$ is the starting change in temperature $\Delta T$ and $\Delta T_{f}$ is the final change in the temperature $\Delta T$ obtained due to the heating process of the laser illumination. In the steady state, there are two temporal regions. In the first one $0 < t < \delta t$ and then $P\left( t \right) = P_{0}$ and in the second $\delta t < t < \Delta t$ then one has $P\left( t \right) = 0$. The integral equation for the first temporal segment is:

87 $$ \mathop \int \limits_{0}^{t} dt = \mathop \int \limits_{{\Delta T_{s} }}^{{\Delta T_{f} }} \frac{C}{{\rho AP_{0} - A\varepsilon \sigma T_{0}^{4} - \left( {4A\varepsilon \sigma T_{0}^{3} + \alpha } \right)\Delta T\left( t \right)}}d\Delta T\left( t \right) $$

which gives the solution of:

88 $$ t = \frac{C}{{ - \left( {4A\varepsilon \sigma T_{0}^{3} + \alpha } \right)}}ln\left| {\frac{{ - \left( {4A\varepsilon \sigma T_{0}^{3} + \alpha } \right)\Delta T_{f} + \rho AP_{0} - A\varepsilon \sigma T_{0}^{4} }}{{ - \left( {4A\varepsilon \sigma T_{0}^{3} + \alpha } \right)\Delta T_{s} + \rho AP_{0} - A\varepsilon \sigma T_{0}^{4} }}} \right| \forall 0 < t < \delta t $$

and in the second it is:

89 $$ \mathop \int \limits_{\delta t}^{t} dt = \mathop \int \limits_{{\Delta T_{f} }}^{{\Delta T_{s} }} \frac{C}{{ - A\varepsilon \sigma T_{0}^{4} - \left( {4A\varepsilon \sigma T_{0}^{3} + \alpha } \right)\Delta T\left( t \right)}}d\Delta T\left( t \right) $$

which gives the solution of:

90 $$ t - \delta t = \frac{C}{{ - \left( {4A\varepsilon \sigma T_{0}^{3} + \alpha } \right)}}ln\left| {\frac{{ - \left( {4A\varepsilon \sigma T_{0}^{3} + \alpha } \right)\Delta T_{s} - A\varepsilon \sigma T_{0}^{4} }}{{ - \left( {4A\varepsilon \sigma T_{0}^{3} + \alpha } \right)\Delta T_{f} - A\varepsilon \sigma T_{0}^{4} }}} \right| \forall \delta t < t < \Delta t $$

The analytical solution reached in this mathematical derivative provides the first ever-analytical solution to the heating produced due to the laser illumination applied on a nano-photonic device while the illumination is produced in a periodic time changing function, e.g. a pulsed illumination.

The main conclusion, that one can reach from the solution obtained in Eqs. (88), (90), is obtained when substituting $t = \delta t$ in Eq. (88) and then extracting the final temperature change $\Delta T_{f}$ obtained due to the heating and then substituting $t = \Delta t$ in Eq. (90) and extracting from it the starting temperature change $\Delta T_{s}$ obtained due to the heating, to be used for the next cycle of laser illumination. From those two equations it is seen that both $\Delta T_{f}$ as well as $\Delta T_{s}$ depend exponentially on $\delta t$ and on $\Delta t$ and therefore those two values can be controlled by the pulsed laser illumination parameters of $\delta t$ and $\Delta t$. Unlike in the CW case where the heating process will continue and the temperature change will continue to increase as long as the laser is turned on, and no control of the final temperature change $\Delta T$, obtained due to the heating, can be obtained for fixed illuminating laser power.

## Fabrication results

### Fabrication process

The starting material is made of a Silicon On Insulator (SOI) wafer with silicon layer thickness of 30 nm and n-type doping of 10^17^ cm^3^. The buried insulator oxide (BOX) layer thickness is 150 nm, and the p-type silicon substrate has a doping concentration of 10^15^ cm^3^. As part of the process flow, thin layers of oxide and nitride are deposited on the SOI wafer. After the photolithography step and etch of the oxide–nitride layers, the surrounding silicon is fully oxidized down to the buried oxide by LOCal Oxidation of Silicon (LOCOS) process. This step creates the desired thick oxide that isolates the channel from the lateral photo-generated current. The next step is to connect the external source and drain voltages to their respective areas in the channel. This connection is ensured by depositing polysilicon layer using a Low Pressure Chemical Vapor Deposition process (LPCVD) followed by doping the poly silicon layer with very high phosphor concentration. Indeed, direct deposition of aluminum on the n-type channel would lead to a non-ohmic Schottky contact. Subsequently, Phosphor doped Silicon Glass (PSG) is grown on the polysilicon layer. Then, a thermal annealing is performed in order to diffuse the phosphor down to the channel through the polysilicon layer, making N + ohmic contact. The V-groove is etched by an anisotropic wet KOH technique.

### Characterization

**Figure 13:**
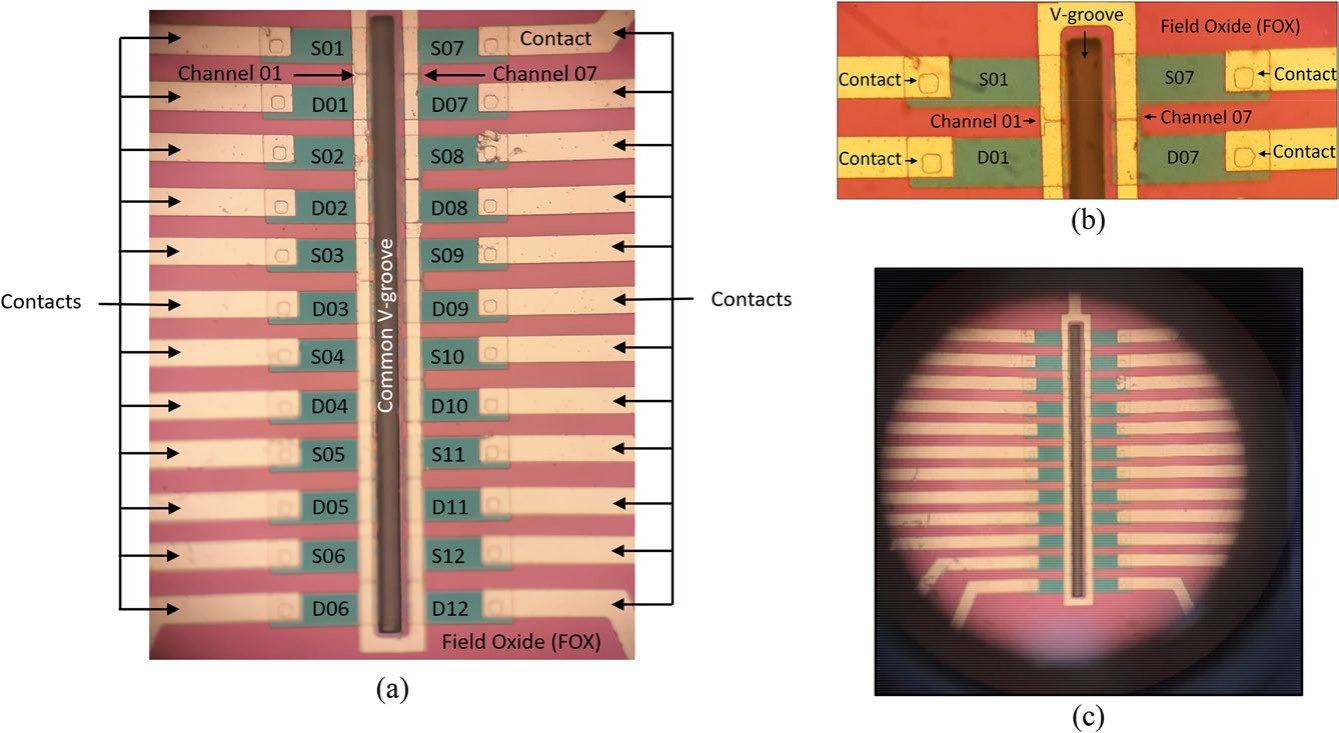
Microscope top views of the final die. (**a**) Array of twelve devices; (**b**) zoom-in of two devices; (**c**) full array of SOIPAMs around common V-groove.

**Figure 14:**
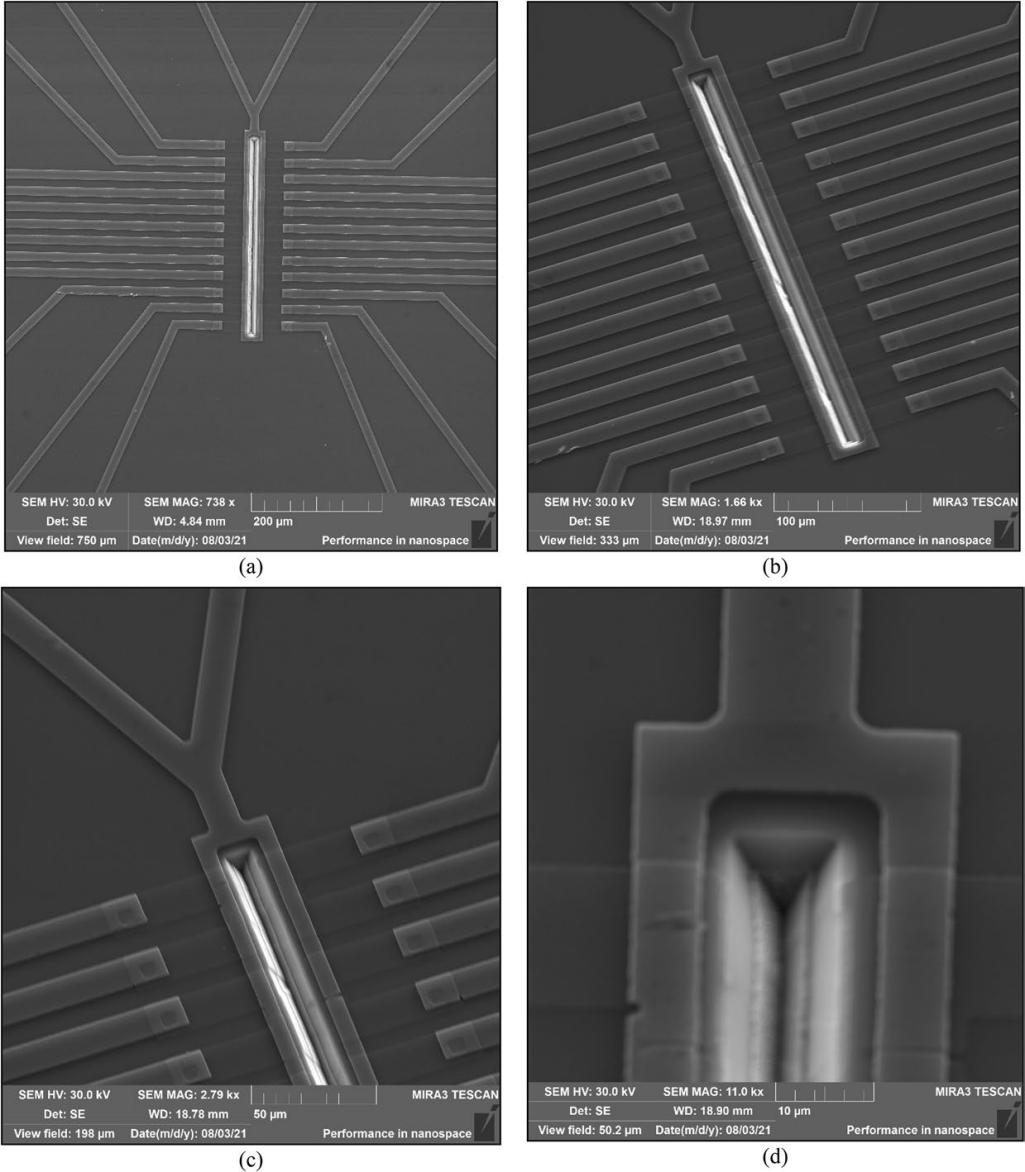
SEM top views of the final die. (**a**) Array of twelve devices and contacts; (**b**) zoom-in of twelves devices around the V-groove; (**c**) zoom-in of the V-groove; (**d**) more zoom-in of the walls of the V-groove.

The preliminary characterization after the processing included visual inspection through a microscope (Fig. 13), and more advanced zoom-in of the device’s structure using Scanning Electron Microscope (SEM), as presented in Fig. 14.

### Preliminary setup

**Figure 15:**
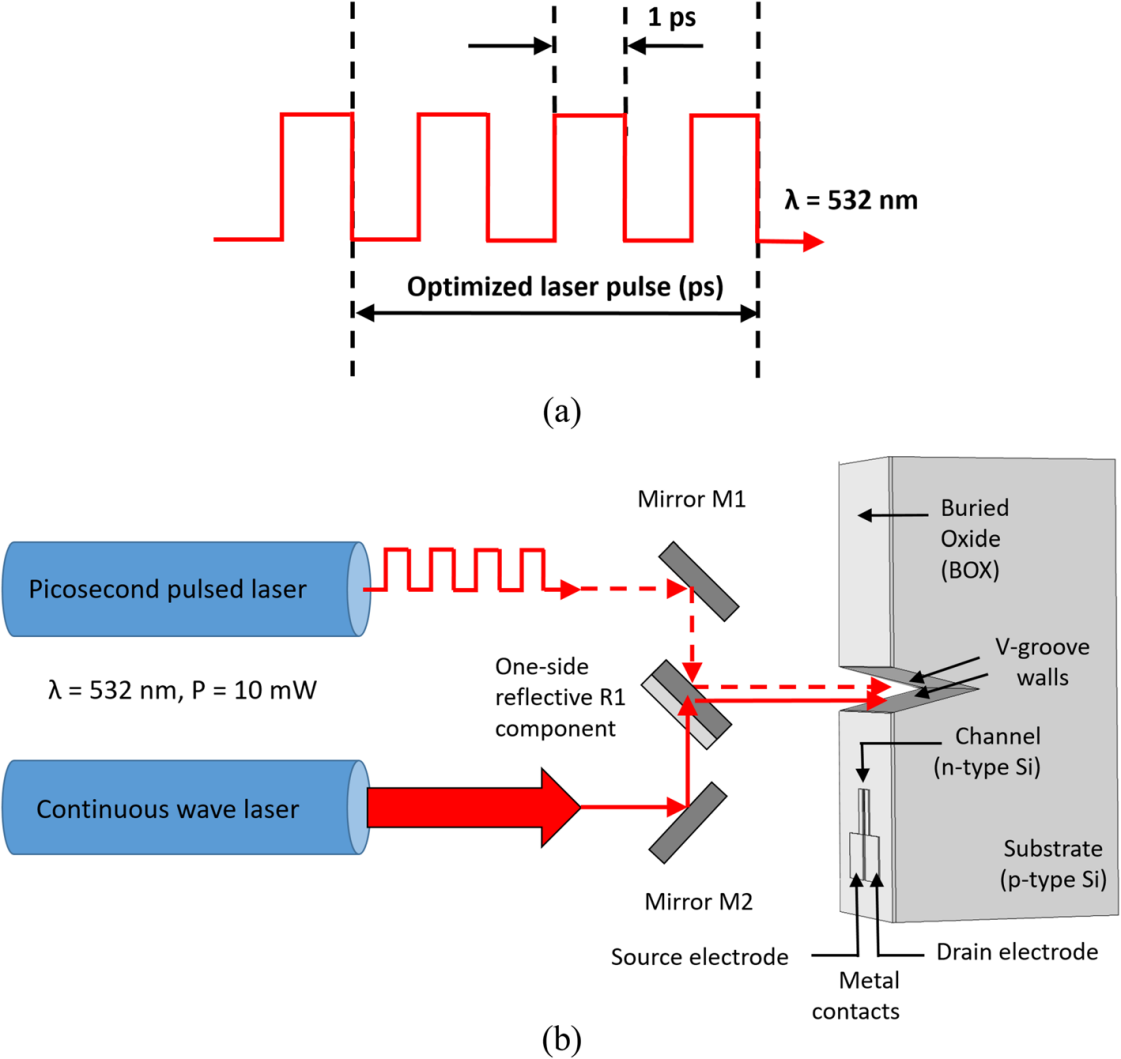
Experimental setup. (**a**) Picosecond pulse diagram; (**b**) setup components.

The preliminary measurement setup is under construction, and it will include two types of lasers. The first one is a picosecond pulsed laser, while the internal pulse of 1 ps (Fig. 15a), and the second one is regular continuous wave (CW) laser (Fig. 15b), illuminating with the same wavelength for comparison. In order to keep a fixed setup, series of mirrors and optics elements are fixed in the path from the illuminating sources to the SOIPAM device.

## Conclusions

In this article, complementary and complex analytical and numerical models are presented for the analysis of the contest between two elementary physical processes, photonic and thermal, inside a SOI photo-activated modulator. It clearly appears that not all the incident-illumination is immediately translated into a generation of electron–hole pairs, since part of the absorbed photons causes self-heating contesting process. A first analytical solution to a complicated partial differential equation, describing the temporal-spatial distribution of free carriers, which are directly associated with solving the localized heating of the proposed device, was presented. Moreover, after establishing this step, a second analytical solution was also presented while using picosecond pulsed laser, in order to prevent unnecessary heating. Such analyses can serve as the basis for the study of additional nanoscale devices.

From this paper, two important conclusions can be drawn regarding the prevention of overheating of semiconductor-based electro-optical devices:

1. Pico-second laser illuminations is effective in preventing energy waste as heating, and in increasing the energy efficiency of illumination to form electron–hole pairs. Of course, this solution is not always applicable, due to the various limitations of such a laser, but when the system allows the usage of this laser and when it requires being effective, it is an optimal solution.
2. The device heats up less when the photons of the illuminating light share energy close to the gap energy between the value level and the conductivity. In such a situation, there is less “excess” energy in the photon absorption so that the system has higher efficiency.

Other methods of heating prevention should be explored in the future for a broader picture of the range of possible solutions.
