# Author Correction: Picosecond pulsed laser illumination: an ultimate solution for photonic versus thermal processes’ contest in SOI photo-activated modulator

**DOI:** 10.1038/s41598-022-07117-3

**Published:** 2022-02-22

**Authors:** David Glukhov, Zeev Zalevsky, Avi Karsenty

**Affiliations:** 1 Advanced Laboratory of Electro‑Optics (ALEO), Department of Applied Physics/Electro‑Optics Engineering, Lev Academic Center, 21 Havaad Haleumi St, POB 16031, 9116001 Jerusalem, Israel; 2 Nanotechnology Educational and Research Center, Lev Academic Center, 9116001 Jerusalem, Israel; 3 Faculty of Engineering, Bar-Ilan University, 5290002 Ramat Gan, Israel; 4 Nanotechnology Center, Bar-Ilan University, 5290002 Ramat Gan, Israel

Correction to: *Scientific Reports* 10.1038/s41598-021-04710-w, published online 28 January 2022

The original version of this Article contained errors.

The paragraph following Eq. 34 was incomplete.

“The numerical solution must meet these two boundary conditions.”

now reads:

“Note that ${x}_{end}$ refers to the end of the semiconductor model, at the insulator layer $({x}_{ins})$. The numerical solution must meet these two boundary conditions.”

Additionally, Eq. 35 was incorrect and contained a duplication of the term ${dx}^{{\prime}}.$

35 $$\frac{{\partial }^{2}\phi }{\partial {x}^{2}}=-\frac{\partial E}{\partial x}=-\frac{\rho }{\epsilon }\Rightarrow \phi (x)-\phi (0)=-\underset{0}{\overset{x}{\int }}Edx=-\frac{1}{\epsilon }\underset{0}{\overset{x}{\int }}\underset{0}{\overset{{x}^{{\prime}}}{\int }}\rho d{x}^{{\prime}}d{x}^{{\prime}}$$

now reads:

35 $$\frac{{\partial }^{2}\phi }{\partial {x}^{2}}=-\frac{\partial E}{\partial x}=-\frac{\rho }{\epsilon }\Rightarrow \phi (x)-\phi (0)=-\underset{0}{\overset{x}{\int }}Edx=-\frac{1}{\epsilon }\underset{0}{\overset{x}{\int }}\underset{0}{\overset{{x}^{{\prime}}}{\int }}\rho d{x}^{{{\prime\prime}}}d{x}^{{\prime}}$$

Lastly, in the Numerical results section, under subheading ‘Identifying optimal parameters’,

“One can see (Fig. 11), that the smaller wavelength the temperature is smaller.”

now reads:

“One can see (Fig. 11), that the longer the wavelength, the smaller the temperature.”

The original Article has been corrected.

